# Knowledge and practice of pediatricians regarding childhood constipation in the Arab world: results from a multicenter survey

**DOI:** 10.1186/s12887-022-03536-3

**Published:** 2022-08-06

**Authors:** Mohammed Hasosah, Azad Haleem, Kevan Jacobson, Bassel Alshemmeri, Aziz Alenazi, Ahmed Abdel Badei, Peggy Massoud

**Affiliations:** 1 Pediatric Gastroenterology Department, King Saud Bin Abdulaziz University for Health Sciences, King Abdullah International Medical Research Center’s (KAIMRC) National Guard Heath Affairs. Hospital, PO Box: 8202, Jeddah, 21482 Saudi Arabia; 2 Pediatric Gastroenterology Department, University of Duhok/College of Medicine, Duhok, Iraq; 3 Pediatric Gastroenterology, British Colombia Children’s Hospital, and British Columbia Children’s Hospital Research Institute, University of British Columbia, Vancouver, BC Canada; 4 Pediatric Gastroenterology Department, Kuwait Oil Company Hospital, Ahmadi, Kuwait; 5 Pediatric Gastroenterology Department, King Saud Bin Abdulaziz University for Health Sciences, King Abdullah International Medical Research Center’s (KAIMRC) National Guard Heath Affairs Hospital, Riyad, Saudi Arabia; 6 Zagazig University, Novalac KSA, Zagazig, Egypt; 7 Saint Joseph University, Novalac MENA Region, Beirut, Lebanon

**Keywords:** Constipation, Knowledge, Practice, Pediatricians, Arab countries

## Abstract

**Objectives:**

We aimed to evaluate knowledge and practice styles among medical providers with different professions and working in different Arab countries regarding their approach to childhood constipation.

**Methods:**

We conducted a cross-sectional multinational survey in eight Arab countries. Pediatric care providers (PCPs), including pediatric specialists (PSs), pediatric residents (PRs), pediatric consultants (PCs), pediatric gastroenterologists (PGs), general practitioners (GPs), and pediatric surgeons (PSu), were included in our study. The survey was anonymous, and participants provided input on the definition and management of constipation.

**Results:**

Of 4000 PCPs, 2579 completed the survey (response rate of 64.5%). Although the majority of respondents were aware of the Rome IV criteria to diagnose constipation, significant differences in the percentage of participants in different geographic countries regarding the definition of constipation were noted. Polyethylene glycol (PEG) was prescribed as a first-line treatment of fecal disimpaction significantly more frequently by pediatricians (PSs, PRs, PCs, PGs) than GPs and PSu (12.8% versus 7.2%, respectively; *p* < 0.001). Additionally, we found that pediatricians used special milk (high magnesium) as a first-choice formula significantly more often than other physicians (53.7% versus 37%, *P* < 0.001). For maintenance therapy, both pediatricians and nonpediatricians used dietary management with a special milk formula more than other treatment options (84.2% and 84%, respectively).

**Conclusions:**

Despite increased awareness of the Rome IV criteria, significant differences in knowledge and practice patterns regarding fecal disimpaction exist among PCPs from different Arab countries. The identification of these gaps may be helpful for policy-makers to produce targeted instructional resources on constipation for PCPs.

## Background

Constipation in children is a growing global public health issue with a prevalence rate ranging between 0.7 and 29.6% [1, 2]. This considerable variation is due to the different diagnostic criteria used to diagnose constipation. Constipation also accounts for 2.5 million physician visits each year, resulting in substantial health care expenses and lowered individuals’ quality of life [3, 4]. Given its consequences on individuals and society, children with constipation who frequently present to the emergency department or are admitted to hospitals for treatment can be effectively managed by pediatric care providers (PCPs), including general practitioners (GPs), family physicians (FPs) and pediatricians, in an outpatient setting [5]. Although constipation is a common pediatric problem, some physicians struggle to manage chronic constipation due to inadequate laxative dosing and maintenance treatment, poor adherence to guidelines, or lack of patient follow-up [6]. A number of internationally accepted standards for symptom-based criteria for the diagnosis of constipation have been developed, such as the Paris Consensus on Childhood Constipation Terminology and the Rome IV criteria. Of these guidelines, none have yet been applied to clinical practice globally. As a result, the North American Society for Pediatric Gastroenterology, Hepatology, and Nutrition (NASPGHAN) established the Constipation Guideline Committee to establish clinical practice guidelines [7].

The cross-sectional survey is one evaluation method used to assess pediatric providers’ knowledge and practice styles (KPS) in clinical practice. Understanding the spectrum of management methods utilized in treating children with constipation would thus be critical for enhancing the quality of life, attaining better health outcomes, and reducing overall health care costs. We previously performed a survey in all regions of Saudi Arabia [8]. We observed considerable disparities in KPS regarding childhood constipation. A survey conducted by Jang et al. [9] also reported discrepancies between actual practice and the Rome IV criteria among pediatricians.

To the best of our knowledge, no previous multicenter survey has been conducted among different Arab countries to assess pediatricians’ KPS regarding childhood constipation. Thus, this study aimed to assess the KPS of different PCPs working in different Arab countries regarding childhood constipation and understand the spectrum of management styles to improve the quality of care and provide better health outcomes.

## Methods

### Study design

We conducted a cross-sectional multinational survey in 8 countries, including Saudi Arabia, Iraq, Lebanon, Oman, Bahrain, United Arab Emirates (UAE), Qatar, and Kuwait. Our study was compliant with the Strengthening the Reporting of Observational Studies in Epidemiology (STROBE) guidelines [10]. These survey questions were built and tested for accuracy and functionality prior to distribution by 2 pediatric gastroenterologists (MH and KJ) who had 10 years of experience at our institution.

### Study participants

In the included countries, physicians listed as PCPs by the health authorities were eligible to be included in this study and classified as follows:

I. Pediatricians, including pediatric specialists (PSs), pediatric residents (PRs), pediatric consultants (PCs), and pediatric gastroenterologists (PGs).
II. Nonpediatricians, including GPs, FPs, pediatric surgeons (PSu), or other physicians who work in the field of pediatric medicine (clinical treatment, research, or education).

Both groups of participants mainly treated infants and children. Based on these criteria, a target population of approximately 19,000 physicians was generated. Of them, 4000 participants were stratified randomly. A minimum sample size of 2120 was calculated using the Raosoft sample size calculator website. However, to attain a more statistically significant representation of the study population, the authors chose a random sample of 4000 participants after stratifying for gender and profession.

### Instruments

The user-friendly survey was created to evaluate the KPS of pediatric care providers in diagnosing and managing childhood constipation. A group of PGs piloted the KPS survey initially built by the 2 pediatric gastroenterologists from King Saud Bin Abdulaziz University for Health Sciences. Then, the survey was revised according to the reproducibility, validity, and question value. A total of 10 PGs and five pediatric practitioners reviewed the original pool of items for content and ease of understanding; based on the findings of the pilot study, modifications and adjustments were performed (Cronbach’s alpha = 0.8). The questionnaire was distributed through direct communication as well as online using Survey Monkey over four months from March 2021 to June 2021. A few participants were questioned in face-to-face interviews utilizing a questionnaire in English. We attached a cover letter along with a summary of the study. The cover letter declared that the questionnaire was anonymous, and the participants’ identities would not be included in the study record. The questionnaire was expected to take approximately 10 min to complete. There was no incentive to participate. The data were manually entered into an Excel sheet. Data entry was undertaken twice, and the two copies were compared.

The questionnaire includes 15 items in 3 subscales:

- Practice and demographic characteristics (5 items):
  1. What is your title?
  2. What is your age?
  3. What is your gender?
  4. What is the country of your practice?
  5. What is the type of your health care institute?
- Prevalence and definition of constipation (5 items) and management of constipation (4 items):
  1. What is the percentage of cases of childhood constipation that you see in your clinic?
  2. Of these cases, what is the percentage of infants with constipation that are younger than 1 year old?
  3. What are the main symptoms that you look for to diagnose constipation in infants and children?
  4. How often do you encounter organic causes of constipation (e.g., congenital malformation of anorectum or spine, Hirschsprung disease, allergy, metabolic/endocrine condition, cystic fibrosis, etc.)?
  5. What is the main concern that parents have regarding their children’s constipation?
  6. In your opinion, what is your first-line treatment for infantile fecal disimpaction (clean-out)?
  7. In your opinion, what type of infant formulas do you usually recommend for the dietary management of infantile constipation?
  8. Do you believe that dietary management with adapted special formulas will help infantile functional constipation?
  9. In your opinion, what is your choice for long-term maintenance therapy of constipation?
- Knowledge level for published guidelines recently on constipation by the European Society for Pediatric Gastroenterology, Hepatology, and Nutrition (ESPGHAN) and NASPGHAN (1 item).

The survey includes multiple-choice questions. The participants were asked to select the correct answer to every question. Responses were measured on a standard scale (sometimes, most of the time, all of the time, seldom, and never). PCPs were asked to provide demographic data and practice characteristics, including gender, age, practice type (subspecialty or general), title, and health care institute level, to identify potential influential differences in the systematic approach when evaluating constipation. We assessed PCPs’ KPS in terms of the definition, diagnosis, and management of pediatric constipation.

### Statistical analysis

For statistical analysis, Stata/IC 12.1–2011 software (StataCorp LP, College Station, TX) was used. Dichotomous data were reported as frequencies and percentages. To compare dichotomous variables, chi-square or Fisher exact tests were used as appropriate. The results were considered significant if *P* < 0.05.

## Results

### Demographic data

Of the 4000 questionnaires distributed to PCPs, 2579 questionnaires were completed and analyzed, yielding a response rate of 64.5%. Most of the participants were males (*n* = 1611; 62.5%), PSs (*n* = 1471; 57%), and between 30 and 50 years of age (*n* = 1679; 65.2%). Most of the participants were from Saudi Arabia (*n* = 1083; 42%) followed by Iraq (*n* = 415; 16.1%) and Lebanon (*n* = 355; 13.8%). The demographic and practice characteristics of the study participants are shown in Table 1.

**Table 1:** Demographic characteristics of respondents and institutions

Variable	Response	No. (%)
**Job title (*n* = 2579)**	Pediatric Specialist	1471 (57%)
	Pediatric Resident	174 (6.7%)
	Pediatric Consultant	455 (17.6%)
	Pediatric Gastroenterologist	47 (1.8%)
	Pediatric Surgeon	35 (1.4%)
	General Practitioner/Family physician	308 (11.9%)
	Other	89 (3.5%)
**Age (*n* = 2579)**	< 30 years old	144 (6.5%)
	30–50 years old	1679 (65.2%)
	> 50 years old	756 (29.3%)
**Gender (*n* = 2579)**	Male	1611 (62.5%)
	Female	968 (37.5%)
**Where is your region of practice? (*n* = 2579)**	Kingdom of Saudi Arabia	1083 (42%)
	Iraq	415 (16.1%)
	Lebanon	355 (13.8%)
	Oman	110 (4.3%)
	Bahrain	151 (5.9%)
	United Arab Emirates	134 (5.2%)
	Qatar	128 (5%)
	Kuwait	203 (7.9%)
**What is the level of your health care institute? (*n* = 2579)**^a^	Governmental	1256 (48.7%)
	Private	1390 (53.9%)
	University	179 (6.9%)

^a^Some participants were working for more than one health care institution

### Definition of constipation

**Fig. 1:**
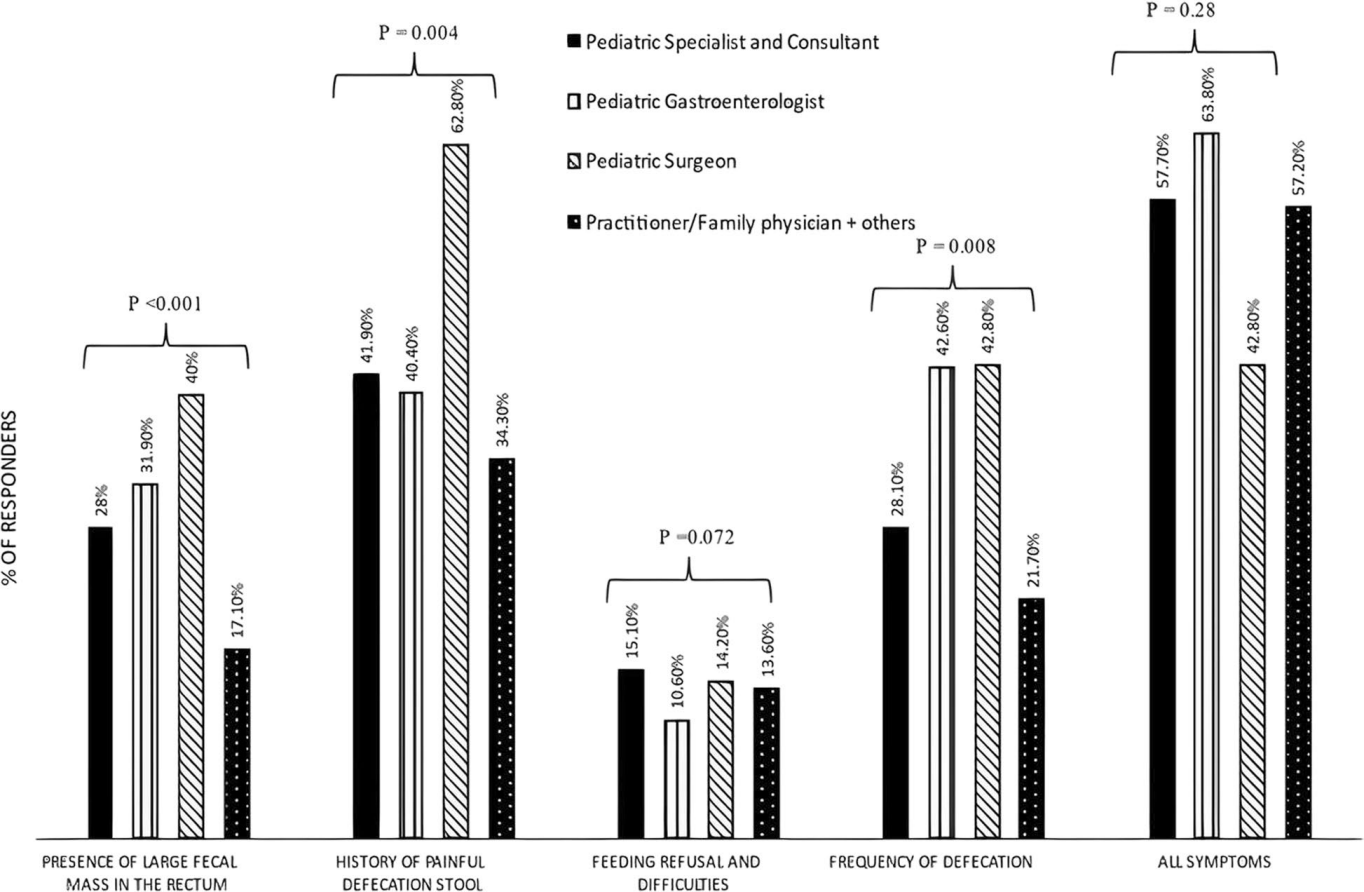
Correlation between the status of respondents and the constipation definition

Regarding the Rome IV criteria to diagnose constipation in infants and children, 2340 respondents (90.7%) were aware of these criteria. In response to the percentage of cases of childhood constipation seen in the clinic, most respondents (*n* = 908; 35.2%) reported a rate of 10–30%. We observed substantial variations in the proportion of physicians with different job titles who defined constipation as the existence of a large fecal mass in the rectum (*P* < 0.001) with the maximum value noted in PSu (*n* = 14; 40%) and the minimum value noted in GPs/FPs (*n* = 52; 17%). Additional questions focused on respondents’ knowledge about the best definition of constipation and asked respondents to choose one of the following: history of painful defecation, decreased frequency of defecation, feeding difficulties, and all symptoms. Considerable differences in the proportion of physicians with different job titles who defined constipation as a history of painful defecation were observed (*P* = 0.004) with the maximum value noted in PSu (*n* = 22; 62.8%) and minimum value noted in GPs/FPs (*n* = 106; 34.3%). Regarding feeding refusal, no significant differences were noted among physicians with different job titles (*p* = 0.07 and *p* = 0.28). Figure 1 shows the relationship between the status of respondents and the constipation definition.

### Differences in management styles between pediatricians and nonpediatricians

Polyethylene glycol-based solutions (PEG) were prescribed by pediatricians as a first-line treatment of fecal disimpaction significantly more often than other PCPs (12.8% versus 7.2%, respectively; *p* < 0.001). Moreover, pediatricians prescribed fleet enemas significantly more than other physicians (9.9% versus 4.4%, respectively; *p* < 0.001). Surprisingly, we found that half of the PCPs (*n* = 239; 55.3%) prescribed special milk formula for the treatment of fecal dis-impaction.

For maintenance therapy of constipation, pediatricians prescribed PEG and lactulose significantly more often than other physicians (17.5% versus 5.6% and 35.3% versus 29.9%, respectively; *P* < 0.001). Although 363 (> 80%) PCPs used dietary management with special milk formula for the maintenance of constipation, no significant differences were found between pediatricians and nonpediatricians (*p* > 0.05). No significant differences were found between pediatricians and nonpediatricians regarding long-term medications, such as stool lubricants and stimulant laxatives (*p* > 0.05). The majority of PCPs believed that dietary management with adapted formulas could help infantile constipation. Responders’ status and practice style regarding the management of constipation are shown in Table 2.

**Table 2:** Responders’ status and practice style regarding the management of constipation

Variable	Items	Pediatric care provider, No. (%)		Total	*P* value
		**Pediatricians**	**Nonpediatricians**		
		**(*n* = 2147)**	**(*n* = 432)**		
**Do you use the Rome IV criteria to diagnose constipation in infants and children?**		1973 (91.9%)	367 (84.9%)	2340	< 0.001
**What is your first-line treatment for fecal disimpaction (clean-out)?**	Dietary management with special milk formula	1059 (49.3%)	239 (55.3%)	1298	
	Glycerin suppository	513 (23.9%)	115 (26.6%)	628	
	Osmotic laxatives (polyethylene glycol-based solutions)	275 (12.8%)	31 (7.2%)	306	
	Fleet Enemas	213 (9.9%)	19 (4.4%)	232	< 0.001
	Magnesium citrate	11 (0.5%)	4 (0.9%)	15	
	Stimulant laxatives	47 (2.2%)	17 (3.9%)	64	
	Others	29 (1.4%)	7 (1.6%)	36	
**What is your choice for long-term maintenance therapy of constipation?**^a^	Lactulose	758 (35.3%)	116 (29.9%)	874	0.001
	Polyethylene glycol-based solutions	375 (17.5%)	24 (5.6%)	399	0.001
	Magnesium hydroxide	82 (3.8%)	19 (4.4%)	101	0.571
	Dietary management with special milk formula	1807 (84.2%)	363 (84.0%)	2170	0.94
	Stool softeners/lubricants (docusate, mineral oil)	243 (11.3%)	55 (12.7%)	298	0.402
	Stimulant laxatives (senna, bisacodyl)	136 (6.3%)	28 (6.5%)	164	0.909
	Others	–-	–-	–-	–-
**What type of infant formulas do you usually recommend for the dietary management of infantile constipation?**^a^	Partially hydrolyzed formula	310 (14.4%)	74 (17.1%)	384	0.152
	Comfort/sensitive formula	548 (25.5%)	143 (33.1%)	691	< 0.001
	High-magnesium formula	1151 (53.6%)	156 (36.1%)	1307	< 0.001
	Formula with pro- and prebiotics	676 (31.5%)	155 (35.9%)	831	0.075
	Other	–-	–-	–-	–-
**Do you believe that dietary management with adapted formulas will help infantile functional constipation?**		2028 (94.5%)	379 (87.7%)	2407	0.051

Dichotomous data are presented as counts and percentages

^a^The included respondents could choose more than one answer

### Practice style of constipation management among different countries

The practice styles of constipation management among the different countries revealed important variations given the lack of published guidelines written in those countries or a lack of knowledge. Lactulose was the most common laxative agent used in Saudi Arabia (*n* = 382, 35.27%), Iraq (*n* = 194, 46.75%), Kuwait (*n* = 52, 25.62%), Oman (*n* = 49, 44.55%), UAE (*n* = 48, 35.82%), and Qatar (*n* = 24, 18.75%) (*P* < 0.001). In contrast, PEG was the most common laxative agent prescribed in Lebanon (28.45%, *P* < 0.001). Regarding dietary management of infantile constipation among 8 countries, PCPs used a high-magnesium formula significantly more often than other formulas in Saudi Arabia (*n* = 524, 48.38%), Kuwait (*n* = 81, 39.90%), Lebanon (*n* = 258, 72.68%), Oman (*n* = 71, 64.55%), Qatar (*n* = 94, 73.44%), UAE (*n* = 72, 53.73%), and Iraq (*n* = 164, 39.52%). Conversely, a partially hydrolyzed formula was the least commonly used formula in all countries (*P* < 0.001). The relationship between the status of respondents and the practice style of constipation management among different countries is shown in Table 3.

**Table 3:** Correlation between responders’ status and countries’ concern about constipation

Variable	Items, No. (%)	Bahrain		Iraq		KSA		Kuwait		Lebanon		Oman		Qatar		UAE		*P* value
		**(*n* = 151)**		**(*n* = 415)**		**(*n* = 1083)**		**(*n* = 203)**		**(*n* = 355)**		**(*n* = 110)**		**(*n* = 128)**		**(*n* = 134)**		
**Do you use the Rome IV criteria to diagnose constipation in infants and children?**		137	90.73%	354	85.3%	977	90.21%	186	91.63%	343	96.62%	97	88.18%	124	96.88%	122	91.04%	< 0.001
**What is your first-line treatment for fecal disimpaction (clean-out)?**	Dietary management with special milk formula	60	39.74%	192	46.27%	538	49.68%	91	44.83%	225	63.38%	43	39.09%	93	72.66%	56	41.79%	< 0.001
	Glycerin suppository	55	36.42%	83	20%	252	23.27%	66	32.51%	69	19.44%	35	31.82%	21	16.41%	47	35.07%	
	Osmotic laxatives (polyethylene glycol-based solutions)	21	13.91%	83	20%	123	11.36%	25	12.32%	22	6.20%	16	14.55%	2	1.56%	14	10.45%	
	Fleet enemas	8	5.30%	25	6.02%	127	11.73%	13	6.40%	32	9.01%	7	6.36%	6	4.69%	14	10.45%	
	Magnesium citrate	2	1.32%	0	0%	8	0.74%	1	0.49%	1	0.28%	0	0%	3	2.34%	0	0%	
	Stimulant laxatives	2	1.32%	23	5.54%	22	2.03%	6	2.96%	2	0.56%	5	4.55%	3	2.34%	1	0.75%	
	Others	3	1.99%	9	2.17%	13	1.20%	1	0.49%	4	1.13%	4	3.64%	0	0%	2	1.49%	
**What is your choice for long-term maintenance therapy of constipation?**^a^	Lactulose	45	29.80%	194	46.75%	382	35.27%	52	25.62%	80	22.54%	49	44.55%	24	18.75%	48	35.82%	< 0.001
	Polyethylene glycol-based solutions	38	25.17%	44	10.60%	114	10.53%	39	19.21%	101	28.45%	11	10.00%	6	4.69%	46	34.33%	
	Magnesium hydroxide	16	10.60%	8	1.93%	31	2.86%	6	2.96%	19	5.35%	6	5.45%	7	5.47%	8	5.97%	
	Dietary management with special milk formula	119	78.80%	316	76.10%	909	83.90%	174	85.70%	333	93.80%	94	85.50%	114	89.10%	111	82.80%	
	Stool softeners/lubricants (docusate, mineral oil)	18	11.92%	90	21.69%	101	9.33%	28	13.79%	20	5.63%	13	11.82%	8	6.25%	20	14.93%	
	Stimulant laxatives (senna, bisacodyl)	4	2.65%	49	11.81%	58	5.36%	13	6.40%	25	7.04%	6	5.45%	7	5.47%	2	1.49%	
	Others	–-		–-		–-		–-		–-		–-		–-		–-		–-
**What type of infant formulas do you usually recommend for dietary management of infantile constipation?**^a^	Partially hydrolyzed formula	34	22.52%	79	19.04%	171	15.79%	30	14.78%	39	10.99%	7	6.36%	7	5.47%	17	12.69%	< 0.001
	Comfort/sensitive formula	62	41.06%	111	26.75%	295	27.24%	78	38.42%	54	15.21%	25	22.73%	17	13.28%	49	36.57%	
	High-magnesium formula	43	28.48%	164	39.52%	524	48.38%	81	39.90%	258	72.68%	71	64.55%	94	73.44%	72	53.73%	
	Formula with pro- and prebiotics	52	34.44%	127	30.60%	338	31.21%	72	35.47%	143	40.28%	22	20.00%	35	27.34%	42	31.34%	0.003
	Other	–-		–-		–-		–-		–-		–-		–-		–-		–-

Dichotomous data are presented as counts and percentages

^a^The included respondents could choose more than one answer

### Physicians’ knowledge of parental/family concerns

Supplementary questions investigated the participants’ parental/family knowledge and concerns regarding constipation consequences as well as predisposing factors. Approximately one-third of families reported no concerns, and the majority of parents reported that they believe constipation is functional in nature. Approximately 14% expressed their concerns that constipation was caused by stenosis/stricture, 37% thought it would last into adulthood, 15% reported that the medications for constipation had adverse events, and only 3% thought that constipation was caused by a malignancy.

## Discussion

To the best of our knowledge, this is the largest study in Middle East and Arab countries describing pediatricians’ knowledge and practice patterns regarding childhood constipation.

Previous studies comprising primary health care providers from different countries documented substantial variations in practice in terms of evaluating and treating childhood constipation according to published clinical guidelines [11–13].

In the present study, we assessed the KPS of PCPs among Arab countries regarding childhood constipation to improve the children’s quality of care. With regard to the awareness and application of the Rome IV criteria, the majority (*n* = 1973; 92%) of our PCPs were aware of these criteria to diagnose constipation in infants and children; the highest percentages of physicians were reported in Qatar (96.88%) followed by Lebanon (96.62%) and Kuwait (91.6%). Lower rates were reported in the UAE, Bahrain, Oman, Saudi Arabia, and Iraq (ranging from 85.3% to 91.04%). This finding could be attributed to the high level of adherence to the NASPGHAN guidelines in Saudi Arabia. We believe that decreased awareness in these countries is due to a lack of clinical practice guideline use in hospitals and that lack of time or rapid access to the electronic database are barriers to practice. Previous studies have found that guideline adherence in medicine is generally low and varies widely across centers [14].

In contrast to our study, a Korean survey reported a lower awareness rate of the Rome IV criteria among pediatricians (16.6%). The authors noted that differences occurred between actual practice and Rome IV standards between PGs and GPs [9]. Scarpato et al. [15] surveyed 278 pediatricians from nine countries and reported that less than 50% of PCPs used the ROME criteria to diagnose functional gastrointestinal diseases, whereas children’s parents used the symptoms to diagnose constipation. Similar findings regarding unfamiliarity with the Rome criteria were reported by Schurman et al. [16]. This finding raises concerns about how to enhance guideline awareness among GPs, who treat most childhood constipation cases. Thus, it is crucial to include formal training and didactic lessons for PCPs, especially GPs and FPs, throughout residency training, active educational resources, and frequent continuous medical education (CME) lectures to improve guideline awareness and adherence.

In the current study, we observed several discrepancies between PCPs (pediatricians and nonpediatricians) regarding the definition and diagnosis of childhood constipation, as shown in Table 2.

Despite increased awareness and application of the Rome IV criteria among PCPs, there are no obvious reasons why PSu differs from FPs/GPs regarding the definition of childhood constipation. However, medical education and adequate disclosure of constipation guidelines are necessary for PCPs.

Given the scarcity of published randomized controlled studies, the management of childhood constipation is primarily based on the physicians’ expertise rather than the published literature [17]. However, the NASPGHAN Constipation Guideline Committee stated that fecal disimpaction is advised before maintenance therapy with oral laxatives with/without rectal laxatives [7]. Rectal enemas or transient high-dose oral PEG for three days are two pharmacological treatments for fecal disimpaction [18, 19]. High-dose PEG is associated with a greater incidence of fecal incontinence throughout management when compared to enemas; nonetheless, PEG is suggested as the primary choice for disimpaction since it may be delivered orally and is less invasive [18, 19]. Surprisingly, we reported in our study that half of the PCPs (50.3%) prescribed a special milk formula (high-magnesium formula) for the treatment of fecal disimpaction. The National Institute for Health and Care Excellence (NICE) guidelines recommended against the use of dietary interventions alone as first-line management for constipation and recommended the use of PEG-based solutions or lactulose with an escalating dose regimen [20]. Magnesium is a safe and effective agent in the treatment of functional constipation that can promote balanced water reabsorption from the large intestine mucosa, retaining the appropriate water amount so that stools have a softer consistency without being loose [21]. Xinias et al. [22] evaluated the efficacy of a synbiotic formula with high magnesium content for maintenance treatment of functional constipation. This formula significantly improved functional constipation, resulting in a better quality of life for the parents and infants. In our study, most PCPs prescribed this formula for disimpaction and maintenance treatment of constipation despite pediatricians reporting using rectal disimpaction (glycerin suppository, fleet enemas, and PEG) significantly more often than nonpediatricians.

Regarding long-term maintenance therapy of constipation, pediatricians prescribed PEG and lactulose more frequently than other physicians. These findings are consistent with the meta-analysis by Lee-Robichaud et al. [23]. The meta-analysis concluded that PEG should be used in preference to lactulose in the treatment of chronic constipation. The majority of our PCPs recommended using dietary management with a special milk formula, mainly magnesium formula, for the long-term maintenance of constipation. Based on pediatricians’ clinical practice reported in Benninga et al. [24], they endorsed the use of a high-magnesium formula as it significantly improved stool consistency. Together with the observation in our PCPs, we suggest that prescribing a high-magnesium formula will significantly improve stool consistency compared with other formulas. A randomized comparator-controlled study of 286 infants was conducted and demonstrated that normal stool consistency occurred significantly more often in children who used the magnesium-rich formula compared to infants fed the control formula (81.8% vs. 41.1%; *p* < 0.001).

Significant differences in the percentage of participants in different geographic regions who prescribed lactulose were noted with maximum levels observed in Saudi Arabia, Iraq, Kuwait, Oman, UAE, and Qatar and a minimum in Lebanon. This finding may be attributed to the relative unavailability of PEG and lactulose.

In terms of participants’ knowledge of parental/family concerns regarding constipation consequences, our findings revealed significant variations in PCPs’ teaching approaches for childhood constipation. No concerns were reported by 33% of participants, and most parents believed that constipation was functional in nature. According to the NICE guidelines, the family should be reassured that the common cause of constipation is functional.

In summary, this is the largest survey study of the KPS of pediatricians and nonpediatricians regarding childhood constipation. The data by Schmulson et al. [25] taken together with the observation in our PCPs suggest that the application of the Rome criteria for constipation was insufficient.

A major strength of our study is that the study is the first multicenter cross-sectional study conducted among many Arab countries comprising a large number of PCPs. However, our study has some limitations. PCPs with a specific interest in constipation were more likely to reply to the questionnaire and consequently may have introduced a response bias. Another limitation of this study is that the data relied on subjective observations made by the participants.

Our survey revealed that NASPGHAN guidelines were poorly adhered to, based on PCPs' responses. NASPGHAN guidelines are systematically developed tools that present recommendations to highly specialized pediatric gastroenterologists. We expect a poor adherence of nonpediatricians due to limited education to promote evidence-based practice.

## Conclusion

Despite increased awareness of the Rome IV criteria to diagnose constipation, significant differences in knowledge and practice patterns exist among PCPs from different Arab countries. In addition, the clinical practice was not consistent among PCPs of the included countries. This discrepancy is related to many factors and is primarily due to exaggeration of the clinical experience that may lead to poor adherence to guidelines and inadequate medical knowledge updating. The identification of these gaps may be helpful for policy-makers to generate targeted instructional resources on constipation for PCPs.

## Data Availability

All data generated or analyzed during this study are included in this published article.

## Abbreviations

PCPs: Pediatric care providers
GPs: General practitioners
FPs: Family physicians
NASPGHAN: North American Society for Pediatric Gastroenterology, Hepatology, and Nutrition
KPS: Knowledge and practice styles
UAE: United Arab Emirates
STROBE: Strengthening the Reporting of Observational Studies in Epidemiology
PSs: Pediatric specialists
PRs: Pediatric residents
PCs: Pediatric consultants
PGs: Pediatric gastroenterologists
PSu: Pediatric surgeons
ESPGHAN: European Society for Pediatric Gastroenterology, Hepatology, and Nutrition
PEG: Polyethylene glycol
CME: Continuous medical education
NICE: National Institute for Health and Care Excellence
